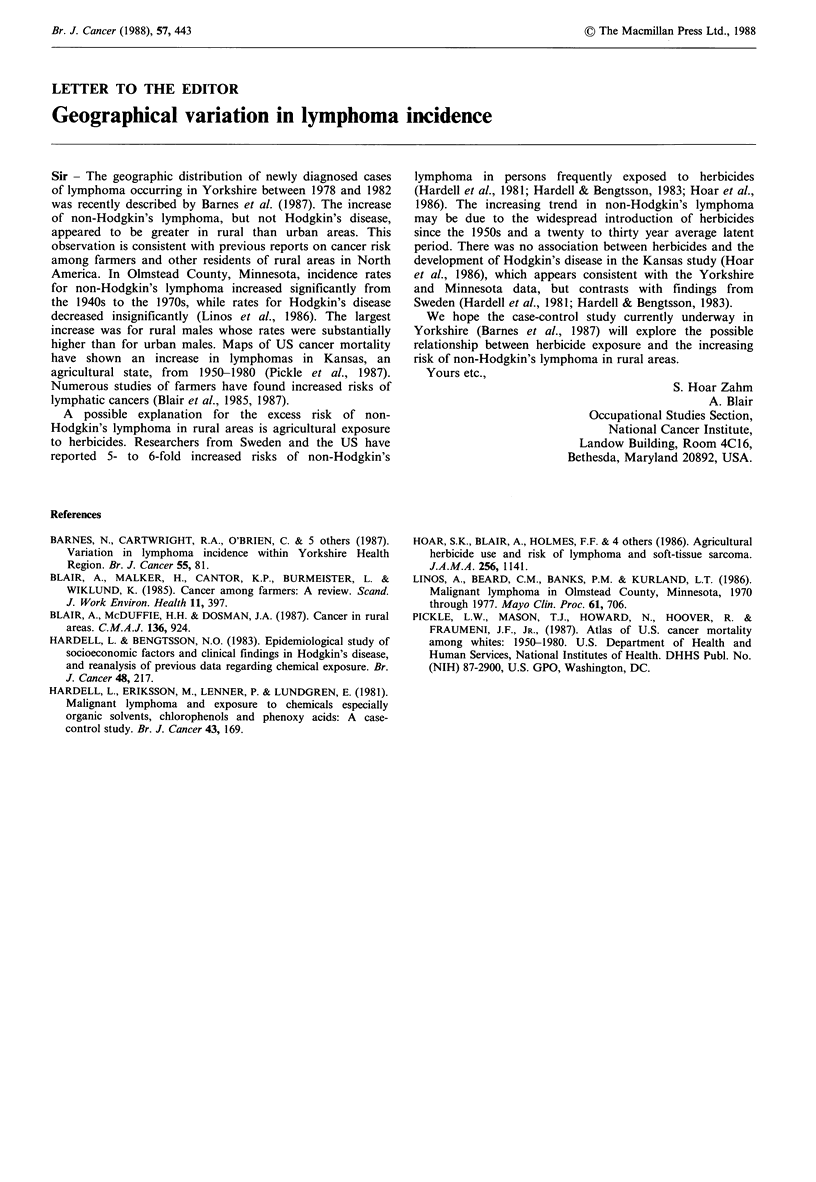# Geographical variation in lymphoma incidence.

**DOI:** 10.1038/bjc.1988.102

**Published:** 1988-04

**Authors:** S. H. Zahm, A. Blair


 © The Macmillan Press Ltd., 1988

LETTER TO THE EDITOR

### Geographical variation in lymphoma incidence

**Sir** – The geographic distribution of newly diagnosed cases of lymphoma occurring in Yorkshire between 1978 and 1982 was recently described by Barnes *et al.* (1987). The increase of non-Hodgkin's lymphoma, but not Hodgkin's disease, appeared to be greater in rural than urban areas. This observation is consistent with previous reports on cancer risk among farmers and other residents of rural areas in North America. In Olmstead County, Minnesota, incidence rates for non-Hodgkin's lymphoma increased significantly from the 1940s to the 1970s, while rates for Hodgkin's disease decreased insignificantly (Linos *et al.*, 1986). The largest increase was for rural males whose rates were substantially higher than for urban males. Maps of US cancer mortality have shown an increase in lymphomas in Kansas, an agricultural state, from 1950–1980 (Pickle *et al.*, 1987). Numerous studies of farmers have found increased risks of lymphatic cancers (Blair *et al.*, 1985, 1987).

A possible explanation for the excess risk of non-Hodgkin's lymphoma in rural areas is agricultural exposure to herbicides. Researchers from Sweden and the US have reported 5- to 6-fold increased risks of non-Hodgkin's lymphoma in persons frequently exposed to herbicides (Hardell *et al.*, 1981; Hardell & Bengtsson, 1983; Hoar *et al.*, 1986). The increasing trend in non-Hodgkin's lymphoma may be due to the widespread introduction of herbicides since the 1950s and a twenty to thirty year average latent period. There was no association between herbicides and the development of Hodgkin's disease in the Kansas study (Hoar *et al.*, 1986), which appears consistent with the Yorkshire and Minnesota data, but contrasts with findings from Sweden (Hardell *et al.*, 1981; Hardell & Bengtsson, 1983).

We hope the case-control study currently underway in Yorkshire (Barnes *et al.*, 1987) will explore the possible relationship between herbicide exposure and the increasing risk of non-Hodgkin's lymphoma in rural areas.

Yours etc.,

S. Hoar Zahm
A. Blair
Occupational Studies Section,
National Cancer Institute,
Landow Building, Room 4C16,
Bethesda, Maryland 20892, USA.

### References

BARNES, N., CARTWRIGHT, R.A., O'BRIEN, C. & 5 others (1987). Variation in lymphoma incidence within Yorkshire Health Region. *Br. J. Cancer* **55**, 81.

BLAIR, A., MALKER, H., CANTOR, K.P., BURMEISTER, L. & WIKLUND, K. (1985). Cancer among farmers: A review. *Scand. J. Work Environ. Health* **11**, 397.

BLAIR, A., McDUFFIE, H.H. & DOSMAN, J.A. (1987). Cancer in rural areas. *C.M.A.J.* **136**, 924.

HARDELL, L. & BENGTSSON, N.O. (1983). Epidemiological study of socioeconomic factors and clinical findings in Hodgkin's disease, and reanalysis of previous data regarding chemical exposure. *Br. J. Cancer* **48**, 217.

HARDELL, L., ERIKSSON, M., LENNER, P. & LUNDGREN, E. (1981). Malignant lymphoma and exposure to chemicals especially organic solvents, chlorophenols and phenoxy acids: A case-control study. *Br. J. Cancer* **43**, 169.

HOAR, S.K., BLAIR, A., HOLMES, F.F. & 4 others (1986). Agricultural herbicide use and risk of lymphoma and soft-tissue sarcoma. *J.A.M.A.* **256**, 1141.

LINOS, A., BEARD, C.M., BANKS, P.M. & KURLAND, L.T. (1986). Malignant lymphoma in Olmstead County, Minnesota, 1970 through 1977. *Mayo Clin. Proc.* **61**, 706.

PICKLE, L.W., MASON, T.J., HOWARD, N., HOOVER, R. & FRAUMENI, J.F., JR., (1987). Atlas of U.S. cancer mortality among whites: 1950–1980. U.S. Department of Health and Human Services, National Institutes of Health. DHHS Publ. No. (NIH) 87-2900, U.S. GPO, Washington, DC.